# Estimating the influence of dietary composition and management on nutrient intake and excretion and methane emission in different pig categories

**DOI:** 10.1371/journal.pone.0323024

**Published:** 2025-05-28

**Authors:** Saman Lashkari, Frederik R. Dalby, Uffe Krogh, Elvira Sattarova, Christian F. Børsting

**Affiliations:** 1 Department of Animal and Veterinary Sciences, Aarhus University, AU Viborg, Research Centre, Foulum, Tjele, Denmark; 2 Department of Biological and Chemical Engineering, Environmental Engineering, Aarhus University, Denmark; 3 SEGES, Agro Food Park 15, Aarhus N, Denmark.; CREA: Consiglio per la ricerca in agricoltura e l'analisi dell'economia agraria, ITALY

## Abstract

The study aimed to estimate the effect of diet composition, pig production stage, in-housing conditions, and manure management on methane (CH_4_) emissions from enteric fermentation, manure stored in the barn, and the outdoor storage tank. For each pig category, an estimation for emissions was made for a standard Danish pig diet based on wheat, barley, and soybean meal. Within each category of pigs, emissions were also estimated for diets with different levels and types of dietary fiber from sugar beet pulp, wheat bran, oats, wheat, or soy hulls, which were included as a partial substitution for wheat or barley. In all diets within four pig categories, feed intake, excreted dry matter, feces mass, and urine volume (g/d per animal) increased in sugar beet pulp, wheat bran, oat, or soy hull diets compared to the average Danish diet. In grower-finisher pigs, the sum of CH_4_ emissions from enteric fermentation, manure stored in the barn, and the outdoor storage tank were 9.8, 10.2, 11.0, 11.0, and 11.2 (kg/year/animal place) for wheat diet, average Danish diet, oat diet, wheat bran diet, and sugar beet pulp diet, respectively, while in gestating sows, were 16.9, 17.5, 18.4, 19.6, 19.7, and 23.2 (kg/year/animal place) in wheat diet, average Danish diet, oat diet, sugar beet pulp diet, wheat bran diet, and soy hull diet, respectively. Contribution of CH_4_ emissions from manure stored in the barn plus outdoor storage tank for the average Danish diet accounted for 95, 90, 83, and 84% of total CH_4_ emissions in weaned pigs, grower-finisher pigs, lactating sows, and gestating sows, respectively. In conclusion, feed composition has a considerable impact on CH_4_ emissions. Enteric CH_4_ and CH_4_ emissions from manure stored in the barn and in the outdoor storage tank were increased by elevated concentration of residual fiber in all four pig categories except for enteric CH_4_ in weaned pigs.

## Introduction

Methane (CH_4_) and nitrous oxide (N_2_O) from agricultural emissions account for one-fourth of the annual increase in radiative forcing leading to climate change [1], and 32% of global anthropogenic CH_4_ originates from agriculture [2]. Pig production is the second largest contributor to global livestock-related greenhouse gas emissions, accounting for 9% [3]. Enteric CH_4_ emissions from pigs account for 1.14% of the global enteric CH_4_ emissions from livestock production, and CH_4_ emissions from pig manure account for 27.4% of the global CH_4_ emissions of livestock manure [4], but this may vary considerably depending on the region and standard practice for manure management.

In regions with intensive livestock production, pig manure is managed as liquid slurry in pits and channels underneath the barn floor [5], which provides an anaerobic environment. In the anaerobic environment, microorganisms convert parts of the organic matter (OM) to end-products CH_4_ and carbon dioxide (CO_2_) through a cascade of biological reactions [5,6]. Therefore, managing slurry from livestock has become of interest to reduce the climate impact from livestock production.

The CH_4_ emission from pig slurry is affected by temperature, slurry removal frequency, and the amount of degradable OM available for anaerobic fermentation [5–9]. The amount of OM available for anaerobic fermentation in the slurry depends on diet composition and digestibility in the pig. It has been well established that dietary composition impacts feed and nutrient intakes [10,11]. Le Goff et al. [10] reported a higher feed intake in adult ovariectomized sows fed a diet containing high dietary fiber (200 g total fiber/kg DM) by the inclusion of maize bran, wheat bran, or sugar beet pulp compared to a wheat-based diet (low dietary fiber diet with 100 g total fiber/kg DM). Similarly, Casas et al. [12] reported a higher feed intake in growing gilts fed diets containing 40% of full-fat rice bran or defatted rice bran compared to corn and soybean meal based diet when fed at 1.5 × the metabolizable energy required for maintenance.

Le Sciellour et al. [13] reported total tract OM digestibility of 88% and 81% in diets containing 15% of wheat bran and 10% soybean hulls in grower-finisher pigs, respectively. Similarly, Le Goff et al. [10] reported that inclusion of high dietary fiber (200 g total fiber/kg DM) from maize bran, wheat bran, or sugar beet pulp reduced OM and crude protein (CP) digestibility compared to a wheat-based diet (low dietary fiber diet with 100 g total fiber/kg DM) in adult ovariectomized sows. The difference between the degradability of OM in the study by Le Sciellour et al. [13] and study by Le Goff et al. [10] is due to different diet and nutrient compositions. Sommer et al. [14] estimated the degradability of OM in fresh excreta to be 89% in pig slurry. Therefore, diet composition has a large impact on the amount of excreted OM and OM composition, which may affect the CH_4_ emission from pig slurry.

Jørgensen et al. [15] reported that enteric CH_4_ emissions (L/d) varied from 0.11–0.15 in weaned pigs, 0.28–12.1 in grower-finisher pigs, and 3.2–28.7 in adult sows, and that feed composition is the most critical factor affecting enteric CH_4_ emission. The authors demonstrated [15] that daily enteric CH_4_ production is highly correlated with total fiber (R^2^ = 0.75) and total fermentable fiber (FF) concentration in the diet (R^2^ = 0.86). Jarret et al. [16] studied the effect of a low fiber diet or a high fiber diet with 15% of dried distiller’s grain on emitted methane from growing pigs and reported a significantly higher CH_4_ emission in manure from pigs fed the high fiber (92 L CH_4_/pig) compared to the control diet (62 L CH_4_/pig) after a 100-day biomethane potential test.

Feed spillage into manure pits is challenging to quantify but could potentially have a high impact on CH_4_ emissions due to the high degradability of feed OM in the manure pit. In a preliminary laboratory study, it has been demonstrated that CH_4_ from pig manure increased by more than 50% over 7 days of manure storage with 5% feed spillage [17]. Ferket et al. [18] estimated that growing pigs spill 1.5 g feed each time it leaves a feeder, and Russell et al. reported that particularly poor trough design can significantly increase feed spillage [19].

Considering the degradability of OM in pig and slurry, it is hypothesized that the dietary nutrient composition will affect the nutrient intake, nutrient excretion, enteric CH_4_, and CH_4_ emissions from manure stored in the barn and the outdoor storage tanks. Diets containing high amounts of non-starch polysaccharides (NSP) and FF are expected to increase the enteric CH_4_ emission, and high residual fiber (RF) content in excreta is expected to increase CH_4_ emission from manure stored in the barn and the outdoor storage tank. Therefore, the objective of the present study was to estimate the effect of different diet compositions on nutrient intake, nutrient excretion, enteric CH_4_ emission, and CH_4_ emissions from manure stored in the barn and outdoor storage tanks for weaned pigs, grower-finisher pigs, gestating sows, and lactating sows. Additionally, the effect of feed spillage levels, as one of the important management factors, on CH_4_ emission from manure stored in the barn and outdoor storage tanks was estimated.

## Materials and methods

The current study had four major aims for estimating the effect of dietary composition: I) on nutrient intake and excretion, II) on enteric CH_4_ emissions, III) on the downstream effect of nutrient composition of slurry (feces + urine) on CH_4_ emissions from manure stored in the barn and the outdoor storage tank, and IV) on the effect of feed spillage levels on CH_4_ emissions from manure stored in the barn and the outdoor storage tank. For the estimation of enteric CH_4_ emissions, the dietary NSP and RF were varied to clarify diet effects on CH_4_ emissions. In the estimation of CH_4_ emissions from manure stored in the barn and the outdoor storage tank, the digestibility of nutrients was calculated and the nutrient composition of slurry and management parameters were used as a determinant factor for methane emission.

### Pig categories

The current estimation was performed in four different pig categories including: 1) weaned pigs, which is defined as pigs with body weight 9–30 kg, 2) grower-finisher pigs, which is defined as pigs with body weight 31–115 kg, 3) gestating sows, and 4) lactating sows.

### Dietary composition of diets

Table 1 shows the diets used for estimating the influence of dietary composition and management on nutrient intake and excretion and CH_4_ emissions in different pig categories. In the current study, the average Danish diet represents the diet commonly used in commercial Danish pig farms [20]. The diet used in the current estimation for weaned pigs was the commonly used Danish diet for weaned pigs from 9–15 kg. To ensure the requirements for weaned pigs (9–15 kg) were fulfilled throughout the 9–30 kg weight interval, we made the simulation based on the diet, which could fulfill the requirements during the most critical phase from 9–15 kg.

**Table 1:** Diet composition of the average Danish diet used for estimation of nutrient intake and excretion and CH_4_ emissions in different pig categories (% DM of diet)^1^.

	Weaned pigs	Grower-finisher pigs	Gestating sows	Lactating sows
**Barley**	20.0	27.0	47.20	18.20
**Wheat**	56.11	40.43	24.49	49.92
**Rye**	0.00	10.00	10.00	10.00
**Sugar beet pulp**	0.00	0.00	2.50	2.50
**Soy hulls**	0.00	0.00	8.10	0.00
**Soybean meal, toasted**	14.00	16.70	5.20	15.30
**Sunflower meal, dehulled**	0.00	2.00	0.00	0.00
**Potato protein**	2.50	0.00	0.00	0.00
**Fish meal**	1.00	0.00	0.00	0.00
**Vegetable oil**	1.80	0.80	0.50	0.50
**L-lysine (70%)**	1.10	0.45	0.00	0.50
**DL-methionine (99%)**	0.23	0.07	0.00	0.10
**L-Threonine (98.5%)**	0.30	0.20	0.00	0.15
**L-tryptophan (98%)**	0.09	0.00	0.00	0.00
**L-valine, (96.5%)**	0.15	0.00	0.00	0.00
**Monocalcium phosphate**	0.30	0.30	0.20	0.80
**Calcium carbonate (36% calcium)**	1.52	1.45	1.25	1.45
**Salt**	0.70	0.40	0.20	0.38
**Vitamin and mineral supplement**	0.20	0.20	0.20	0.20
**Energy content per kg as fed**				
**FUsv** ^**a**^	112	107	–	–
**FUso** ^**b**^	–	–	99	105

^a^ FUsv = Feed Unit for growing pig [21].

^b^ FUso = Feed Unit for sows [21].

^1^ Details of diets used for estimation of nutrient intake and excretion and methane emission in different pig categories are shown in the Supporting information S1, S2, S3, and S4 Tables.

In order to obtain different sources and levels of RF in the diet and excreta, 5% of wheat was replaced by 5% of sugar beet pulp or 5% of wheat bran, whereas 10% of wheat was replaced by 10% of oats in weaned pigs (S1 Table), grower-finisher pigs (S2 Table), and lactating sows (S3 Table). Furthermore, 10% of barley was replaced by 10% of wheat to obtain a diet with a low level of RF and FF. Thus, in the weaned pigs, grower-finisher pigs, and lactating sows, five different diets were used for estimation of nutrient intake and excretion and CH_4_ emissions: 1) average Danish diet, 2) average Danish diet with 5% of wheat replaced by 5% sugar beet pulp 3) average Danish diet with 5% of wheat replaced by 5% of wheat bran, 4) average Danish diet with 10% of wheat replaced by 10% of oats, and 5) average Danish diet with 10% of barley replaced by 10% of wheat.

A similar approach was followed in gestating sows (S4 Table); however, the difference was that 10% of wheat was replaced by 10% of sugar beet pulp, wheat bran, oats, or soy hulls. Furthermore, 10% of barley was replaced by 10% of wheat to obtain a diet with a low level of RF and FF. Thus, for the gestating sows, six different diets were used for estimation of nutrient intake and excretion and CH_4_ emissions: 1) average Danish diet, 2) average Danish diet with 10% of wheat replaced by 10% sugar beet pulp, 3) average Danish diet with 10% of wheat replaced by 10% of wheat bran, 4) average Danish diet with 10% of wheat replaced by 10% of oats, 5) average Danish diet with 10% of barley replaced by 10% of wheat, and 6) average Danish diet with 10% of wheat replaced by 10% of soy hulls.

In all diets, feed ingredient composition was slightly adjusted to meet the nutrient requirements for different pig categories. After the partial substitution of new feedstuffs, all the manipulated diets were supplemented with crystalline amino acids, calcium, phosphorous, and vitamin and mineral supplements to meet the requirements for different pig categories [22]. The nutrient composition of diets for four pig categories was calculated based on the nutrient concentration of different feed ingredients used for the simulation (Table 2) according to the following equation:

**Table 2:** Nutrient composition of feedstuffs used for estimation of dietary composition effects on feed intake, feces composition and CH_4_ emissions in four pig categories^a^.

		Nutrient composition (g/kg of DM)^b^								
	DM (g/kg)	OM	CP	CF	Starch	Sugar	RF	iNDF	NSP	sNSP
**Barley**	854	979	109	31	604	21	214	28	186	56
**Sugar beet pulp**	898	946	88	16	0	59	783	34	700	290
**Fish meal**	929	848	772	98	0	0	0	0	0	0
**Oats**	853	974	114	59	471	18	313	118	232	39
**Potato protein**	900	977	859	22	0	0	96	1	0	0
**Rye**	857	984	95	20	613	32	224	29	152	43
**Soy hulls**	875	950	108	24	7	19	792	10	680	126
**Soybean meal, toasted**	876	926	487	29	33	93	284	6	217	63
**Vegetable oil**	995	1000	0	1000	0	0	0	0	0	0
**Sunflower meal**	900	925	388	27	17	56	437	156	255	10
**Wheat**	856	983	113	24	670	19	157	22	119	29
**Wheat bran**	874	936	168	52	220	37	459	97	374	30

^a^ Data obtained from Danish table values [23].

^b^ OM; organic matter, CP; crude protein, CF; crude fat, RF; calculated residual fiber, iNDF; indigestible neutral detergent fiber, NSP; non-starch polysaccharides, sNSP; soluble non-starch polysaccharides.


$$X_{i,k}=\sum_{j}^{nk}a_{j,k}x_{i,j}$$ (1)

where X_i,k_ denotes *i *= 1, 2, …, 11 is the calculated concentration of either DM, ash, OM, CP, crude fat (CF), starch, sugar, RF, indigestible neutral detergent fiber (iNDF), NSP, or soluble NSP (sNSP) in the *k*th diet (g/kg feed for DM and g/kg DM diet for other nutrients, respectively); n_k_ = number of ingredients in the *k*th diet; a_j,k_ denotes the proportion of the *j*^th^ feed ingredient in the *k*th diet (kg DM feed ingredient/kg DM diet), and x_i,j_ denotes the *i*^th^ concentration of either DM, ash, OM, CP, CF, starch, sugar, RF, iNDF, NSP, or sNSP in *j*^th^ feed ingredient (g/kg DM feed ingredient). The values for DM, ash, OM, CP, CF, starch, and sugar concentration in different feed ingredients were obtained from Danish feed tables [23], and values for NSP and sNSP were obtained from Knudsen [24]. The iNDF concentration in feed ingredients was obtained from the Norfor feed tables [25].

The RF concentration (g/kg DM) was calculated according to Equation (2).


$$\begin{array}{c} \text{RF\textbackslash{}, = \textbackslash{}, OM\textbackslash{} –\textbackslash{} CP\textbackslash{} –\textbackslash{} CF\textbackslash{} –\textbackslash{} starch\textbackslash{} –\textbackslash{} sugar} \end{array}$$ (2)

It is important to note that there are various approaches to analyze the cell wall content of feedstuffs, i.e., dietary fiber, crude fiber [26], natural detergent fiber [27], acid detergent fiber [27] or NSP [28], which are often reported in the literature [10–13,29]. In the present study, the cell wall content was not analyzed; however, the estimated RF values reflect, to some extent, the crude fiber, natural detergent fiber, or acid detergent fiber content in different feedstuffs. Therefore, in the present study, literature values on the effects of crude fiber, neutral detergent fiber, acid detergent fiber, and NSP on nutrient intake, and methane emissions were compared to the results of the present study.

### Feed intake

The intake of each diet for weaned pigs and grower-finisher pigs was calculated using the Danish average growth per batch cycle of pigs [30], specific nutrient content and feed units (FU) of the diet as in Equation (3):


$$\begin{array}{cc} & \text{Feed\textbackslash{} intake\textbackslash{} (kg\textbackslash{} as\textbackslash{} fed/day/pig)}=\text{gain\textbackslash{} (kg\textbackslash{} gain/pig/batch\textbackslash{} cycle)}\\ & \;\times \text{feed\textbackslash{} efficiency\textbackslash{} (FU/kg\textbackslash{} gain) }\times \text{ feed\textbackslash{} per\textbackslash{} FU\textbackslash{} (kg\textbackslash{} feed/FU)/duration\textbackslash{} of\textbackslash{} batch\textbackslash{} cycle\textbackslash{} (days)} \end{array}$$ (3)

The intake of each diet for sows feed was calculated as in Equation (4):


\[Feed\ intake\ (kg\ as\ fed/day/sow)=FU\ (FU/year/sow)/ 365 ×feed\ per\ FU\ (kg\ feed/FU)\] (4)

The intake of FU for sows (FU/year/sow) was estimated by subdividing the different stages of the sow production cycle into those taking place in the gestation and farrowing sections (lactating sows). For gestating sows these stages/phases include feed intake for gilts (104 FU/year/sow), intake before gestation (58.8 FU/year/sow), other intake in gestation section (208 FU/year/sow), intake during pregnancy (582 FU/year/sow), and intake by boar (20.4 FU/year/sow). These stages occur over an estimated 285 feeding days/year/sow. For lactating sows the phases were the weeks before farrowing (39.1 FU/year/sow) and the time after farrowing until weaning (499.3 FU/year/sow), which occur over an estimated 80 feeding days/year/sow.

### Feces and urine excretion

In order to calculate nutrient excretion in feces, the apparent total tract digestibility coefficients for OM and CP for each feedstuff were obtained from National Research Institute for Agriculture, Food and the Environment (INRAE) feed tables [31] for grower-finisher pigs and sows, respectively. The digestibility coefficients obtained for grower-finisher pigs [31] were used for the calculation of OM and CP digestibility in weaned pigs and grower-finisher pigs, and total tract digestibility coefficients obtained for sows [31] were used for the calculation of OM and CP total tract digestibility in lactating and gestating sows. The CF digestibility coefficients for each feedstuff were also obtained from the INRAE tables, and the same digestibility coefficients were used for all four pig categories. The digestibility coefficient for the sum of starch and sugars was calculated [22], and the same values were used for all four pig categories. The digested amount of each nutrient fraction (g/kg of DM) was calculated by multiplying the apparent total tract digestibility coefficient of each nutrient fraction by the amount of OM, CP, CF, and starch plus sugar provided by individual feedstuffs, respectively.

The excreted amount of each nutrient per feedstuff was calculated by subtracting the digested amount (g/kg of DM) from the amount of each nutrient in the diet (g/kg of DM). Afterward, the amount of digested RF (g/kg DM) was calculated as in Equation (5):


$$\begin{array}{cc} \text{Digested\textbackslash{} RF} & =\text{digested\textbackslash{} OM\textbackslash{} –\textbackslash{} digested\textbackslash{} CP}–\text{digested\textbackslash{} CF}\\ & \;–\text{ digested\textbackslash{} starch\textbackslash{} plus\textbackslash{} sugar\textbackslash{} (g/kg\textbackslash{} of\textbackslash{} DM\textbackslash{} for\textbackslash{} each\textbackslash{} nutrient)} \end{array}$$ (5)

The iNDF content was obtained from the Norfor feed tables [25] and is defined as the part of the fiber in the diet that is undegradable. Hence, iNDF is used to partition excreted RF content into an undegradable and a potentially degradable part, where the latter was calculated as in Equation (6):


\[Potentially\ degradable\ RF\ (g/kg\ of\ DM)=RF\ (g/kg\ of\ DM)\ –\ iNDF\ (g/kg\ of\ DM)\] (6)

The potentially degradable RF fraction was used as an input for estimating CH_4_ emission from manure stored in the barn and the outdoor storage tank.

Prapaspongsa et al. [32] estimated the excretion of urine nitrogen according to Equation (7), which was used for grower-finisher pigs, gestating sows, and lactating sows:


\[Urine\ nitrogen\ (g/day)=−21.20 + 0.134 dietary\ CP\ (g/kg)+10.15 DM\ intake\ (kg/d)\] (7)

However, the above equation overestimated the excretion of urine nitrogen in weaned pigs. Therefore, the urine nitrogen excretion for weaned pigs was calculated by subtracting nitrogen in feces and body weight gain (kg/d) from total nitrogen intake [30]. The proportion of urea nitrogen out of total urine nitrogen was estimated as 75% ± 6% (means ± SD) based on the average of three studies [33–35].

In order to calculate the feces mass excretion from grower-finisher pigs, gestating sows and lactating sows, Equation (8) was used [32].


Feces mass (kg/d= 5.405 − 6.31 × digestibility coefficient of OM + 0.505 × DM intake (kg/d) (8)

However, Equation 8 did not fit to the estimated feces mass in weaned pigs, therefore, feces mass for weaned pigs was instead calculated based on the amount of excreted feces DM and a constant of 25% ± 0.67 (DM in feces; mean ± standard deviation of (159 individual weaned pigs) [36]. Urine production for weaned and growing pigs was estimated as 2.0 kg of urine per kg DM intake based on the average value reported by Prapaspongsa et al. [32], whereas urine production was 2.5 kg per kg DM intake in lactating and gestating sows [30]. Volatile fatty acids concentration (VFA) in feces was set to 2.87 ± 0.98 g per kg of excreted feces mass (means ± SD; unpublished data).

### Emission of CH_4_ from enteric fermentation and manure

Enteric CH_4_ (L/d) from weaned pigs and grower-finisher pigs [37] was estimated according to Equation (9).


$$Enteric\;{CH}_{4}\;(L/d=\;–\;0.62\;+\;0.032\;\times \;sNSP\;intake\;(g/d)\;+\;0.025\;\times \;body\;weight\;(kg)$$ (9)

In gestating and lactating sows, enteric CH_4_ was estimated according to Jørgensen et al. [15] as in Equation (10):


$$Enteric\;{CH}_{4}\;(L/d=\;0.440\;+\;0.0206\;\times \;FF\;intake\;(g/day;\;n\;=137,\;R^{2}=\;0.74)$$ (10)

The FF [15] was calculated as in Equation (11).


\[FF=digested\ OM – digested\ CP – digested\ CF – digested\ starch\ and\ sugar\ (g/kg\ of\ DM\ in\ feed\ for\ all\ variables)\] (11)

The CH_4_ emission from the manure was calculated using the Anaerobic Biodegradation Model (ABM) [5,6] with the R-package “ABM” v. 2.0. The ABM model simulates hydrolysis and fermentation of the above-mentioned OM components to VFA and subsequent methanogenesis of VFA to CH_4_ and CO_2_. Hydrolysis and fermentation of OM components follow first order degradation kinetics with temperature sensitive rate constants derived from [7]. However, the model does not consider that OM components may degrade at different rates depending on diet and botanical origin [38]. To demonstrate the link between the digestibility of nutrients and ABM, Equation (12) shows the rate of change (*dCP/dt*, g chemical oxygen demand/day) of CP as an example:


$$\mathrm{\backslash [}\frac{dCP}{dt}=CP_{in}-\;\alpha \times CP\;-\;R\;\times CP\mathrm{\backslash ]}$$ (12)

Where *CP*_*in*_ (g chemical oxygen demand/day) is the CP excreted by the pigs as predicted by the digestibility of nutrients, *α* (1/day) is the hydrolysis/fermentation rate constant, which depends on manure temperature and the OM component, *R* (1/day) is the respiration rate in the manure surface, which depends on manure temperature [39], and CP (g COD) is the amount of CP in the slurry storage/pit. Simultaneously, while CP is degraded, VFA is produced. Production of VFA is more complex and follows stoichiometry described in detail previously [39].

The model is dynamic and simulates the growth of methanogens and the VFA uptake rate of three methanogen populations which are also responsive to temperature and inhibitors in the manure environment. The model parameter values are all accessible from the ABM R-package v. 2.0 with parameter set v2.0 which is available from http://github.com/AU-BCE-EE/ABM.

Input variables that reflect the in-house management of manure and animals differed for the four pig categories simulated in this study. The most important differences were: manure production rate, manure removal frequency from the barn to the outside storage tank, manure temperature in the barn, floor type and surface area of the floor and the manure pits inside the barn, and the chemical composition of the freshly excreted feces OM. These parameters are shown in Table 3 for the different pig categories, except for the feces OM composition. Manure removed from the barn was assumed to be directly transferred to the outside manure storage, which was identical for all pig categories. The outside manure storage tank was 5.5 m high and 36 m in diameter. The outside manure storage was with a tent cover, which reduces ammonia loss and avoids intake from rainfall and water evaporation from the manure in the simulation. Manure was removed from the storage for field application according to the average Danish application pattern for pig farmers [40], where most of the manure is removed in the spring, and a smaller amount is removed in the autumn. This results in an average manure retention time of ~ 4.5 months in the storage tank. The monthly average slurry temperature and slurry mass in a storage tank are provided in the Supporting S5 Table.

**Table 3:** Animal, barn and management characteristics for the four pig categories used for estimation.

	Weaned pigs	Grower-finisher pigs	Gestating sows	Lactating sows
Section type	50% slatted + 50% solid flooring	67% slatted floor, 33% drained floor ^a^	Loose sows, 39% slatted floor, 61% solid floor	Boxes with 50% slatted floor, 50% solid floor
**Manure temperature, °C**	22	18.6	18.6	20
**Manure removal frequency, days**	27	7	30	36
**Floor area/per animal, m** ^**2**^ **/animal place**	0.3	0.65	2.14	4.9
**Manure pit area/ animal, m** ^**2**^ **/animal place**	0.18	0.715	1.05	2.94
**Cycle period, days** ^**b**^	59	89	119	40
**Section empty time, days/cycle**	5	5	0	4
**Washing and spilled water, kg/animal place/cycle**	15	75	0	340
**Average body weight, kg**	18.9	73	245	245

^a^ A drained floor is a slatted floor with smaller and fewer perforations than a normal slatted floor. Floor type affects manure pit area.

^b^ Cycle period includes time the pig is in the section and the time needed to wash the section before the next pig enters or time that the section is empty for other reasons.

### Feed spillage

In order to simulate production conditions, 2% of feed intake was included as feed spillage directly into the manure pits. Thus, the contribution of each nutrient from feed spillage was calculated and added to the manure composition. Furthermore, to evaluate the effect of feed spillage amount on CH_4_ emissions from manure stored in the barn and outdoor storage tank, a simulation was run with 0, 2, 4, 6, and 8% of feed spillage for all four pig categories for the average Danish diet.

## Results

### Feed intake and diet composition

Estimation of dietary composition effect on feed intake, dry matter, and nutrient intake in four pig categories is presented in Table 4. In all four pig categories, feed intake was increased in sugar beet pulp, wheat bran, and oat diets in order to obtain the same daily intake of FU as in the average Danish diet to compensate for the lower energy density in these diets due to a higher content of RF. In contrast, in the wheat diet, both RF content in the diet and feed intake were decreased. In all diets within a pig category, both the CP and CF content of diets remained similar. For gestating sows, feed intake and RF content were highest in the soy hull diet. Feed intake (g/d) of the average Danish diet for different pig categories was estimated in the following order (highest to lowest): lactating sows > gestating sows > grower-finisher pigs > weaned pigs.

**Table 4:** Estimation of dietary composition effect on feed intake and nutrient intake in four pig categories.

			Nutrient composition of diet (g/kg DM)							
	Feed intake (g/d)	DM (g/kg)	CP	CF	Starch	Sugar	sNSP	RF	iNDF	ash
**Weaned pigs**										
Average Danish diet	725	869	201	46	494	28	36	153	18	61
Sugar beet pulp diet ^a^, 5%	747	871	200	45	460	30	39	184	19	62
Wheat bran diet ^a^, 5%	741	870	204	47	472	29	36	164	22	63
Oat diet ^a^, 10%	742	868	201	49	474	28	37	159	28	61
Wheat diet ^b^, 10%	719	869	201	45	500	28	33	148	18	61
**Grower-finisher pigs**										
Average Danish diet	2475	865	181	35	496	33	42	176	23	57
Sugar beet pulp diet ^a^, 5%	2558	867	180	34	462	35	45	207	24	58
Wheat bran diet ^a^, 5%	2536	866	184	36	474	34	42	187	27	58
Oat diet ^a^, 10%	2546	865	181	38	476	33	43	181	33	57
Wheat diet ^b^, 10%	2456	865	181	34	502	33	39	171	23	56
**Lactating sows**										
Average Danish diet	6384	865	169	30	505	32	41	182	20	52
Sugar beet pulp diet ^a^, 5%	6523	867	168	30	472	34	44	213	21	53
Wheat bran diet ^a^, 5%	6514	837	172	32	483	33	41	193	24	54
Oat diet ^a^, 10%	6495	864	170	34	488	32	42	188	30	50
Wheat diet ^b^, 10%	6354	865	170	30	511	32	38	176	20	53
**Gestating sows**										
Average Danish diet	3452	861	125	32	508	26	54	237	24	42
Sugar beet pulp diet ^a^, 10%	3620	865	122	31	443	30	60	300	25	42
Wheat bran diet ^a^, 10%	3598	863	126	35	472	27	53	258	32	43
Oat diet ^a^, 10%	3568	861	128	35	483	26	55	244	33	42
Wheat diet ^b^, 10%	3424	862	123	31	518	25	51	231	23	41
Soy hull diet ^a^, 10%	4199	863	120	32	451	25	63	301	23	41

^a^ Wheat was substituted with equal amounts of sugar beet pulp, wheat bran, oats, and soy hulls, respectively.

^b^ 10% of barley in the average Danish diet was substituted with wheat.

### Feces composition and excretions

Estimation of dietary composition effect on feces composition, amount of excreted DM, feces mass, and excreted urine are presented in Table 5. For all diets in all four pig categories, RF content (g/kg of DM) in feces increased in sugar beet pulp and wheat bran diets compared to the average Danish diet. Similarly, the amount of excreted DM in feces, feces mass, and excreted urine was increased in sugar beet pulp, wheat bran, and oat diets compared to the average Danish diet. However, in the wheat diet, RF and FF content (g/kg of DM) in feces, the amount of excreted DM in feces, feces mass, and excreted urine were reduced compared to the average Danish diet. In gestating sows, RF and FF content (g/kg of DM) in feces, excreted DM, feces mass, and excreted urine were the highest in the soy hull diet. The amount of excreted DM in feces from pigs fed the average Danish diet for different pig categories was predicted in the following order (from highest to lowest): lactating sows > gestating sows > grower-finisher pigs > weaned pigs. Feces mass from pigs fed average Danish diets varied from 326 in weaned pigs to 2582 (g/d per animal) in lactating sows.

**Table 5:** Estimation of dietary composition effect on feces composition, feces mass, urine volume, and feces DM excreted in four pig categories.

	Feces composition (g/kg of DM)						Excretion (g/day/animal)		
	CP	CF	RF	iNDF	Starch	FF	Feces DM	Feces mass	Urine volume
**Weaned pigs**									
Average Danish diet	212	165	358	143	2.8	106	81	326	1259
Sugar beet pulp diet ^a^, 5%	209	154	375	139	2.8	133	89	357	1300
Wheat bran diet ^a^, 5%	204	152	367	154	3.4	111	93	373	1289
Oat diet ^a^, 10%	187	145	361	185	2.4	104	98	390	1289
Wheat diet ^b^, 10%	216	171	345	145	2.9	105	77	307	1249
**Grower-finisher pigs**									
Average Danish diet	216	145	353	163	3.5	125	307	1021	4281
Sugar beet pulp diet ^a^, 5%	212	136	369	159	3.5	151	336	1107	4435
Wheat bran diet ^a^, 5%	208	135	362	172	4.0	130	349	1140	4390
Oat diet ^a^, 10%	192	129	356	199	3.0	122	364	1181	4402
Wheat diet ^b^, 10%	220	149	341	166	3.7	124	291	973	4248
**Lactating sows**									
Average Danish diet	202	182	305	186	3.8	148	609	2582	13806
Sugar beet pulp diet ^a^, 5%	197	174	320	185	3.8	177	647	2677	14148
Wheat bran diet ^a^, 5%	193	168	317	197	4.3	154	696	2720	14101
Oat diet ^a^, 10%	172	159	314	231	3.1	147	732	2745	14035
Wheat diet ^b^, 10%	207	189	289	191	3.9	146	572	2534	13746
**Gestating sows**									
Average Danish diet	145	148	424	160	1.6	175	439	1458	7439
Sugar beet pulp diet ^a^, 10%	140	139	439	160	1.8	132	488	1587	7832
Wheat bran diet ^a^, 10%	137	133	425	181	2.7	185	540	1680	7765
Oat diet ^a^, 10%	131	135	415	194	1.5	176	510	1611	7623
Wheat diet ^b^, 10%	145	152	419	163	1.5	172	417	1413	7382
Soy hull diet ^a^, 10%	129	133	481	134	1.8	220	611	1909	9062

^a^ Wheat was substituted with equal amounts of sugar beet pulp, wheat bran, oats, and soy hulls, respectively

^b^ 10% of barley in the average Danish diet was substituted with wheat

### Emission of CH_4_

Enteric CH_4_ emission and CH_4_ emissions from manure stored in the barn and the outdoor storage tank are presented in Table 6. Enteric CH_4_ emission (g/d) was highest in the soy hull diet in gestating sows (11.93), and highest in the sugar beet pulp diet for weaned pigs (0.48), grower-finisher pigs (3.26), and lactating sows (11.86) and was lowest for wheat diet for all four pig categories. In general, daily enteric CH_4_ emission in the four pig categories was predicted in the following order for the average Danish diet (from highest to lowest: g/d): lactating sows (9.70)> gestating sows (7.90)> grower-finisher pigs (2.56)> weaned pigs (0.33). For the average Danish diet, we estimated 2.29, 1.52, 1.03, and 0.45 (g CH_4_/kg feed intake) in gestating sows, lactating sows, grower-finisher pigs, and weaned pigs, respectively. The proportion of enteric CH_4_ out of total CH_4_ emissions varied among the different pig categories and for the average Danish diet, it accounted for 17, 16, 10, and 5% in gestating sows, lactating sows, grower-finisher pigs, and weaned pigs, respectively.

**Table 6:** Estimation of dietary composition effect on CH_4_ emissions from enteric, manure stored in the barn, and outdoor storage tank in four pig categories.

			CH_4_ emission, kg/year/animal place		
	Enteric CH_4_, g/d/animal	Enteric CH_4_, g/kg of feed intake	Enteric	Manure barn	Manure storage
**Weaned pigs**					
Average Danish diet	0.33	0.45	0.12	1.07	1.42
Sugar beet pulp diet ^a^, 5%	0.48	0.64	0.17	1.10	1.56
Wheat bran diet ^a^, 5%	0.33	0.44	0.12	1.11	1.59
Oat diet ^a^, 10%	0.33	0.44	0.12	1.11	1.59
Wheat diet ^b^, 10%	0.29	0.41	0.11	1.06	1.34
**Grower-finisher pigs**					
Average Danish diet	2.56	1.03	0.93	1.60	7.61
Sugar beet pulp diet ^a^, 5%	3.26	1.27	1.19	1.60	8.28
Wheat bran diet ^a^, 5%	2.63	1.04	0.96	1.60	8.40
Oat diet ^a^, 10%	2.67	1.05	0.97	1.60	8.37
Wheat diet ^b^, 10%	2.45	1.00	0.89	1.62	7.27
**Lactating sows**					
Average Danish diet	9.70	1.52	3.54	9.67	10.86
Sugar beet pulp diet ^a^, 5%	11.86	1.82	4.33	9.83	11.54
Wheat bran diet ^a^, 5%	10.32	1.58	3.77	10.14	12.11
Oat diet ^a^, 10%	9.81	1.51	3.58	10.10	11.94
Wheat diet ^b^, 10%	9.59	1.51	3.50	9.44	10.24
**Gestating sows**					
Average Danish diet	7.90	2.29	2.89	5.76	8.82
Sugar beet pulp diet ^a^, 10%	10.94	3.02	3.99	5.74	9.86
Wheat bran diet ^a^, 10%	8.67	2.41	3.17	5.90	10.59
Oat diet ^a^, 10%	8.09	2.27	2.95	5.81	9.68
Wheat diet ^b^, 10%	7.72	2.26	2.82	5.69	8.35
Soy hull diet ^a^, 10%	11.93	2.84	4.35	5.89	12.94

^a^ Wheat was substituted with equal amounts of sugar beet pulp, wheat bran, oats, and soy hulls, respectively

^b^ 10% of barley in the average Danish diet was substituted with wheat.

The CH_4_ emission from manure stored in the barn were highest for the soy hull diet in gestating sows, wheat bran diet in lactating sows, and wheat bran and oat diets in weaned pigs. However, for grower-finisher pigs, CH_4_ emission from manure stored in the barn were the same in the sugar beet pulp diet, wheat bran diet, oat diet, and average Danish diet. Summation of CH_4_ emissions from manure stored in the barn and outdoor storage was higher in the soy hull diet (only simulated in gestating sows), sugar beet pulp diet, wheat bran diet, and oat diet compared to the average Danish diet in all four pig categories. In contrast, the summation of CH_4_ emissions from manure stored in the barn and outdoor storage was lower in the wheat diet compared to the average Danish diet for all four pig categories.

The CH_4_ emitted from manure stored in the barn and outdoor storage tank in different pig categories was predicted in the following order (from highest to lowest), lactating sows > gestating sows > grower-finisher pigs > weaned pigs, and it was 21, 15, 9.2, and 2.5, respectively (kg CH_4_/year/animal place) for the average Danish diets.

**Fig 1:**
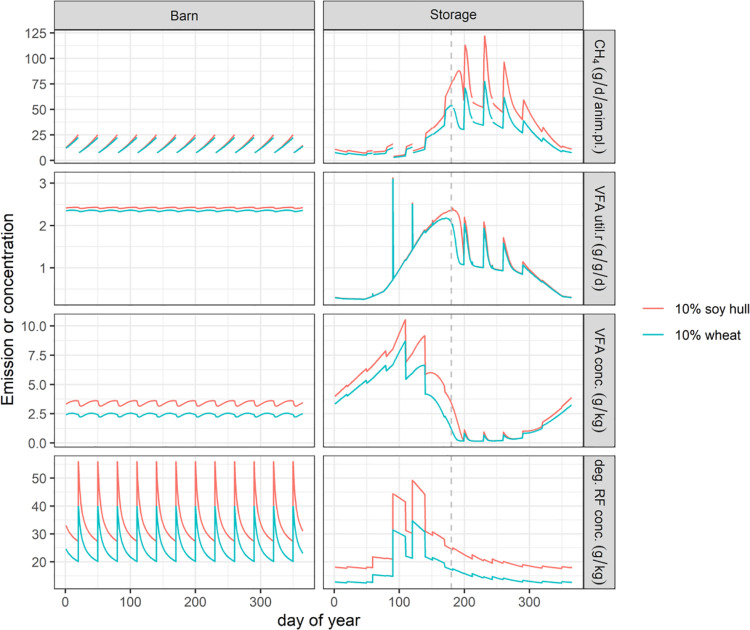
Estimation of CH_4_ emission for the manure and transformation dynamics of substrates in the manure during storage in the barn and in the outdoor storage for gestating sows fed the 10% wheat diet and the 10% soy hull diet. The estimation is for gestating sows with two different diets. CH_4_ is methane, anim.pl. is animal place, VFA is volatile fatty acids, VFA util.r is the utilization rate of VFA by methanogens in g VFA utilized pr. g DM of active methanogen biomass pr. day. Methanogen biomass is assumed to have a stoichiometric composition of C_5_H_7_O_2_N. deg. RF is potentially degradable RF (residual fiber).

The rate-limiting factor influencing CH_4_ emissions is exemplified in Fig 1 for gestating sows fed the 10% wheat diet (high digestibility and lowest DM excretion in feces) and the 10% soy hull diet (low digestibility and highest DM content in feces). A noticeable decline in CH_4_ emission per day from the outdoor storage tank is predicted around day 180, which corresponds to the end of June (Fig 1, dashed gray line) for the 10% wheat diet, whereas CH_4_ emission from the 10% soy hull diet still increases for another ~20 days.

### Effect of feed spillage on barn and storage CH_4_ emission

**Fig 2:**
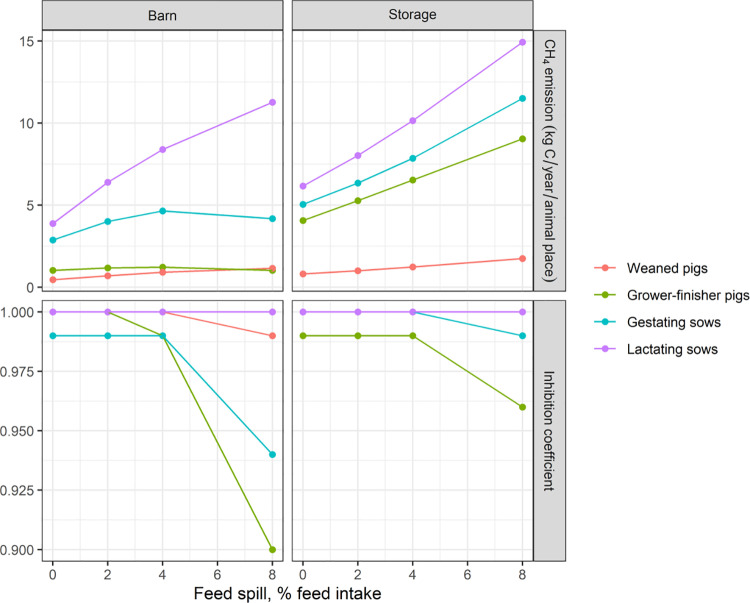
Estimation of CH_4_ emissions and inhibition of methanogens in manure caused by different levels of feed spillage in different pig categories. Simulations were done with the average Danish diet. An inhibition factor of 1 is equivalent to no inhibition of CH_4_ emission and 0 is complete inhibition of methanogens.

Fig 2 illustrates the simulated impact of 0–8% of feed intake as feed spillage. The effect of feed spillage on CH_4_ emissions can be very high, in fact, for lactating sows, the emission from the barn was increased by 65% and in the storage by 30% with just 2% feed spillage, and the increase was extreme if the spillage came up to 8%, namely an increase of 190% in the barn and 142% in the storage. For the other pig categories, the emission increase due to 2% feed spillage was not as profound and ranged from 14–52% from the barn with weaned pigs having the largest relative increase, and 25–29% from the outdoor storage with grower-finisher pigs having the largest relative increase.

## Discussion

### Feed and nutrient intake

The model was developed based on equal net energy (FU) intakes irrespective of diet. Hence, due to the lower energy concentration in sugar beet pulp, wheat bran, oat, and soy hull diets, feed intake was estimated to increase in all four pig categories to compensate for the energy dilution caused by the higher RF content, regardless of the pig category. Our estimation for increased feed intake is supported by previous findings, where it has been found that pigs increase feed intake in response to the lowered energy content of the diet to obtain the energy required for maintenance and growth [41,42]. In addition, both the RF and sNSP intake increased as a result of increased feeding levels in combination with the higher inclusion rate of fiber-rich feed ingredients in the sugar beet pulp, wheat bran, oat, or soy hull diets [11,12,43].

### Feces composition and excretion

As the RF intake increased in all four pig categories, the DM excretion also increased in sugar beet pulp, wheat bran, oat, and soy hull diets compared to the average Danish diet. The higher DM excretion in the sugar beet pulp and wheat bran diets can be explained by the reduced digestibility caused by the higher RF content diets. In agreement with our results, Jarret et al. [16] reported that excreted feces DM increased when growing pigs were fed a diet containing high fiber diet with 15% of dried distiller’s grain compared to a diet with low fiber (395 and 246 g/pig/day of excreted feces DM; respectively). Our results are supported by Le Sciellour et al. [13] who reported that OM digestibility decreased from 88% to 81%, by inclusion of 15% of wheat bran and 10% soybean hulls in the diets of grower-finisher pigs. Inclusion of wheat bran and soybean hulls resulted [13] in increased crude fiber concentration in the diets from 2.8% to 7.7%, while the average feed intake remained the same. The reduced OM digestibility and increased DM excretion may be attributed to the following factors: the increased bulkiness and viscosity of the digesta, which dilute the nutrient concentration and impair the enzymatic hydrolysis and absorption of nutrients [44,45]; the shortened gastrointestinal transit time, which reduces the exposure of the diet to intestinal digestive enzymes [46]; and the increased endogenous losses of energy, protein, and minerals due to higher secretion and excretion of digestive fluids and mucins [44,45]. Furthermore, the increased DM excretion in sugar beet, wheat bran, oat, or soy hull diets compared to the average Danish diet and wheat diet can be explained by the higher concentrations of the fiber that are more resistant to microbial fermentation in the hindgut of pigs [47]. This is in agreement with previous findings, where feeding pigs with diets high in fiber increased the amount of DM excreted per day compared to low fiber diets [43,48]. The current study showed that by increasing the RF intake, the excretion of RF increased due to higher DM excretion and higher RF content in feces. Similar to our results, Jarret et al. [16] reported that the crude fiber excretion increased when growing pigs were fed a diet containing high fiber diet with 15% of dried distiller’s grain compared to low fiber diet (84 and 45 g/pig/day, respectively).

### Enteric CH_4_ emission

Enteric CH_4_ emission was highest for the soy hull diet in gestating sows and sugar beet diet for weaned pigs, grower-finisher pigs, and lactating sows. The level of sNSP in diets of weaned pigs and grower-finisher pigs and level of FF in diets of lactating and gestating sows is the determinant factor for the enteric CH_4_ emission because more substrate is available for microbial fermentation in the hindgut of pigs [49]. In addition, different RF levels explain the differences in enteric CH_4_ emission between different diets within pig categories [49], which is also a reflection of different sNSP levels in diets of weaned pigs and grower-finisher pigs and FF levels in lactating and gestating sow diets. In agreement with our findings, grower-finisher pigs fed with diets containing either 22% sugar beet pulp or 20% soy hulls had a higher enteric CH_4_ emission than those fed with a corn-soybean meal diet [50]. Le Goff et al. [10] investigated the effects of feeding a wheat-based diet (low dietary fiber diet with 100 g total fiber/kg DM) and increased dietary fiber (200 g total fiber/kg DM) by the inclusion of maize bran, wheat bran, or sugar beet pulp to adult ovariectomized sows. The authors reported [10] enteric CH_4_ emissions of 6.1, 11.1, and 9.9 L/d in sows fed wheat-based, maize bran, and sugar beet pulp diets, respectively, which is consistent with our estimation.

Daily enteric CH_4_ emissions were greater in gestating and lactating sows than in grower-finisher pigs and weaned pigs. A higher capacity to ferment fiber in the hindgut, a higher feed intake, and a higher FF intake explain the higher enteric CH_4_ emission in adult sows compared to grower-finisher pigs. Jørgensen et al. [51] and Jørgensen et al. [15] concluded that more substrate is fermented in the large intestine of adult sows than the grower-finisher pigs, resulting in higher enteric CH_4_ emission. The current study revealed that lactating sows emit more enteric CH_4_ than gestating sows, even though the latter have a higher FF concentration in the diet. This is attributed to the lactating sows’ feed intake, which is more than double that of gestating sows on a daily basis, resulting in a greater daily intake of FF.

Our results showed a higher enteric CH_4_ emission in gestating sows compared to lactating when it is expressed per kg of feed intake. In the present study, the higher dietary RF concentration in gestating sow diets partly explains the increased enteric CH_4_ emissions per kg feed intake observed in gestating sows compared to lactating sows. Furthermore, it is well established [52,53] that higher feed intake leads to an increased passage rate of digesta in sows. Consequently, the lower CH_4_ emissions per kg of feed intake observed in lactating sows can also partly be explained by the higher feed intake, and thereby, the higher passage rate of digesta compared to gestating sows. In the present study, the higher CH_4_ emissions per kg of feed intake observed in gestating sows with a fiber-rich diet may also be due to the water-holding capacity of dietary fiber. The increased water retention increases the surface area within the digesta, promoting microbial fermentation of FF [54,55].

### Emission of CH_4_ from manure stored in the barn and outdoor storage tank

The composition of the diet within each pig category showed only a small impact on the simulated emissions from manure stored in the barn. This lack of influence can be attributed to methanogen activity being the primary rate-limiting factor for CH_4_ production from manure rather than the substrate available for the methanogens, which is VFA released from degraded nutrients. This model-based observation aligns with recent empirical findings that reported elevated concentrations of VFA in a pilot-scale pig-house and yet observed comparatively low CH_4_ emissions [8]. However, CH_4_ emissions from manure stored in the barn and outdoor storage tank showed substantial variation across different pig categories in the present study.

The CH_4_ emission per animal place per year from manure stored in the barn was considerably higher for lactating and gestating sows than for grower-finisher and weaned pigs. The elevated excretion of DM and RF, resulting from increased feed intake and RF intake, and the longer retention time of manure in the barn provide an environment for methanogenic growth and consequently considerably higher CH_4_ emission in lactating and gestating sows than the grower-finisher pigs and weaned pigs during both barn and storage periods. The CH_4_ emission from the outdoor storage accounted for most of the manure CH_4_ emission for all pig categories. However, for grower-finisher pigs, the CH_4_ emissions from the outdoor storage tank accounted for 74–77% of total CH_4_ emission, whereas CH_4_ emission from manure stored in the barn accounted for only 15–17% (the rest was enteric CH_4_), which is related to the shorter manure retention time inside the barn (removal interval 7 days). Conversely, for lactating sows, CH_4_ from manure stored in the barn accounted for 44–47% of total CH_4_ emission due to more OM being converted inside the barn with manure removal only every 36 days after each lactation round and a slightly higher in-house manure temperature of 20 °C versus 18.6 °C in the grower-finisher and gestating sows simulations.

Within all pig categories, the increased CH_4_ emissions from the manure stored in the barn and outdoor storage tank from pigs fed sugar beet pulp, wheat bran, and oat diets indicated that the elevated RF content in feces was the primary driver, providing a fermentable substrate for methanogenesis during manure storage in the barn and outdoor storage tank. In agreement with our findings, in growing pigs, there was a 66% increase in CH_4_ (L/pig) in slurry (feces + urine) from pigs fed a diet containing 15% of dried distiller’s grain compared to a control diet with low fiber after 100 days of biomethane potential test due to higher crude fiber excretion [16].

By expressing CH_4_ emissions in the barn per kg of DM excreted, CH_4_ emissions were lowest and highest in grower-finisher pigs and lactating sows, respectively, corresponding to the frequency of manure removal from the barn. These results demonstrate the importance of frequency of manure removal from the barn, and extending the retention time of manure inside a barn increases total manure CH_4_ emission (from barn storage + outdoor storage tank) due to the increased retention time at a higher temperature within the barn. The lowest CH_4_ emissions from manure storage in the barn and highest CH_4_ emission from the outdoor storage tank per excreted DM were found in grower-finisher pigs where CH_4_ emission was shifted from the barn to the outdoor storage tank due to the short manure retention time inside the barn. The higher CH_4_ emission from manure stored in the barn in lactating sows compared to gestating sows could be attributed to the higher barn temperature and the greater amount of DM excreted by lactating sows compared to gestating sows. Furthermore, larger DM excretion in lactating sows also increases the CH_4_ emission from the storage tank. The highest CH_4_ emission from manure stored in the barn was observed in the lactating sows due to higher DM excretion, demonstrating the importance of feed intake and digestibility of nutrients on the barn and storage emissions. Dalby et al. [8] measured 2.35 g CH_4_/pig/day from a grower-finisher pig house with weekly manure removal, which after correction for empty days is 0.81 kg/year/anim. place. This is half of our estimate from the average diet. However, Jørgensen et al. [56] reported 11.6 g/pig/day (enteric + manure emission) from a commercial Danish grower-finisher pig-house with weekly manure removal, which after correction for empty days and enteric emission is around 3 kg/year/anim. place, being approximately twice as high as our estimate. The large variation in measured emission between pig categories and farms was discussed in Dalby et al. [8], but due to many variables that affect emission, it is unclear how much of the variation is due to differences in diet, manure, and animal management.

Across all pig categories, the CH_4_ emissions from the outdoor storage tank in the wheat diet were consistently lower than those associated with other diets. This was attributed to the reduced DM amount in the manure transferred to the outdoor storage tank. The noticeable decline in CH_4_ emission from the outdoor storage tank can be attributed to a decrease in the VFA utilization rate as VFA concentrations approach the half-saturation constant of 0.3–0.6 g VFA/kg. The production rate of VFA follows first-order hydrolysis kinetics and thus is proportional to the OM concentration. This provides a clear illustration of the influence of DM availability for microbial fermentation in the storage and, thereby, affecting CH_4_ emission dynamics.

As stated earlier, the model does not consider that OM fractions may degrade at different rates depending on botanical origin [38]. For example, the FF and sNSP intake, which affect enteric CH_4_ [51], is likely to affect manure CH_4_ production. Jarret et al. [16] reported a 76% increase in manure CH_4_ produced per pig receiving a diet with 20% dried distiller’s grain in slaughter pig slurry incubated in laboratory chambers for 16 days. However, CH_4_ per g of DM excreted was only increased by 7%, suggesting that excretion rates of DM are more complex than assumed in the model applied here [16]. A more systematic evaluation of degradation kinetics and dependence on diet composition is needed.

VanderZaag et al. [57] measured volume-scaled averages of 6.0, 1.8, and 2.1 g CH_4_/m^3^/day over three consecutive years from a Canadian manure storage tank receiving manure from a pig-house with ~2200 grower-finisher pigs. Kupper et al. [58] reported an average baseline emission of 16.3 g CH_4_/m^3^/day from manure storages with pig manure, and Vechi et al. [59] measured an annual average of 37.4 ± 22.3 g CH_4_/m^3^/day from 6 Danish pig manure storage tanks. In comparison, our estimate from the average grower-finisher diet is 7.61 kg/year/anim. place equivalent to 24.4 g/m^3^/day, which is above average but within the 95% confidence bounds reported by Kupper et al. [58]. Our relatively high estimate can be explained by the fact that with only 7 days between manure removals in the grower-finisher pig-house simulation, a high share of the organic matter is transferred to the outdoor storage leading to high CH_4_ emission. The studies included in Kupper et al. [58] are from different countries where manure removal from the pig-house is less frequent than 7 days, whereas some of the farms included in the study by Vechi et al. [59] were with weekly manure removal. However, the studies on manure storage emission are scarce of information about the in-house diet composition and manure management, which limits further comparisons on the effect of particularly diet composition on methane emission in the storage.

### Effect of feed spillage on barn and storage emission

Emission levels were found to be particularly responsive to feed spillage, a source rich in easily degradable sugars and starch that contributes to the manure composition. Notably, the influence of feed spillage on CH_4_ emissions was more pronounced for lactating sows due to the higher feed intake, prolonged manure retention time in the barn, and elevated temperature conditions within the barn environment.

The high nutrient content in the feed, consisting of mostly readily degradable components, suggests that spillage could theoretically lead to a significant increase in CH_4_ emissions, especially if the substrate is the rate-limiting factor for CH_4_ production. This phenomenon is prominently observed in the model simulations with sows where the manure retention time in the barn is high. A 2% feed spillage increases CH_4_ emission in the barn from the manure by 49% in a lactating sow section. Intriguingly, increased feed spillage did not increase CH_4_ emission in the barn for grower-finisher pigs. This is attributed to the inhibition of methanogens induced by elevated concentrations of VFAs (Fig 2. lower panel with inhibition coefficients). The increased VFA concentrations are a consequence of the hydrolysis of starch and sugars from the feed, modeled using the high hydrolysis rate constant for quickly degradable substrates as previously defined in Dalby et al. [7]. The shorter manure retention time and, hence, lower methanogen population for grower-finisher pigs result in reduced VFA utilization [19], thereby exacerbating the inhibitory effect. This nuanced interplay underlines the importance of manure management and its influence on CH_4_ emission dynamics in different pig production stages.

In pig nutritional studies, the apparent feed intake is often reported, which is the sum of actual feed intake and feed spillage. Russell et al. [60] reported high feed spillage in liquid feed trials of weaned pigs due to poor trough design. This was inferred from visual inspection and feed conversion ratios. Ferket et al. [18] postulated that growing pigs spill 1.5 g feed each time it leaves a feeder, which is equivalent to 90 g feed per day, assuming 60 visits to the feeder per day. Based on that postulate and a daily feed intake of approximately 2.86 kg/pig/day for grower-finisher pigs, a feed spillage of ~ 3% is estimated. Feed spillage depends on whether a wet or dry feed system is used and the design of the feeder and trough, and therefore, a large variation in feed spillage can be expected in different pig production systems. The presented simulations suggest that feed spillage should be reduced not only to increase economic aspects of pig production but also to reduce CH_4_ emission.

## Conclusion

The present estimation showed that feed composition, specifically RF concentration, significantly impacts the amount of excreted DM and feces composition. By substituting wheat with sugar beet pulp, wheat bran, or oats in the diet, enteric CH_4_ emission increased due to increased sNSP or FF except for weaned pigs. Also, CH_4_ emission from manure stored in the barn and outdoor storage tank was increased due to increased RF in these diets. Increasing feed spillage due to poor management potentially increases the risk of increased CH_4_ emission from manure stored in the barn and outdoor storage tank. The CH_4_ originating from the manure with outdoor storage emission accounted for 47–74% and the manure in the barn accounted for 16–39% of total CH_4_ emission (sum of enteric and manure derived). Enteric CH_4_ emissions accounted for 5–17% of total CH_4_ emissions. The variation between emissions from manure from different pig categories was attributed to different manure retention times and temperatures inside the pig house. This study suggests that a strategy focusing on both feed composition and manure management is necessary to mitigate CH_4_ emission effectively at the farm-scale level.

## Supporting information

S1 Table Diet composition used for estimation of nutrient intake and excretion and CH_4_ methane emissions in weaned pigs (% of DM). (ZIP)

S2 Table Diet composition used for estimation of nutrient intake and excretion and CH_4_ methane emissions in grower-finisher pigs (% of DM). (ZIP)

S3 Table Diet composition used for estimation of nutrient intake and excretion and CH_4_ methane emissions in lactating sows (% of DM). (ZIP)

S4 Table Diet composition used for estimation of nutrient intake and excretion and CH_4_ methane emissions in gestating sows (% of DM). (ZIP)

S5 Table Monthly average slurry temperature in the outdoor storage used for all modelling scenarios. The slurry mass in the outdoor storage depends on the slurry production and transfer of slurry from the barn slurry pits to the outdoor storage and the application pattern of slurry from the storage to the field. In the table below, the slurry mass in the storage is shown for a farm with 1000 gestating sows and for two different diets. The removal pattern of slurry is identical for all pig categories.
(ZIP)
