# Supplementary material for: Estimating the influence of dietary composition and management on nutrient intake and excretion and methane emission in different pig categories
Source: PLoS One. 2025 May 28;20(5):e0323024. doi: 10.1371/journal.pone.0323024 (PMC12119022; doi:10.1371/journal.pone.0323024)
Supplement: S1 Table — (ZIP) [file pone.0323024.s001.zip › Supporting information_Table_5.docx]

**S5 Table. Monthly average slurry temperature in the outdoor storage used for all modelling scenarios. The slurry mass in the outdoor storage depends on the slurry production and transfer of slurry from the barn slurry pits to the outdoor storage and the application pattern of slurry from the storage to the field. In the table below, the slurry mass in the storage is shown for a farm with 1000 gestating sows and for two different diets. The removal pattern of slurry is identical for all pig categories.**

| **Month** | **Diet** | **Temperature, ℃** | **Slurry mass, tones** |
| --- | --- | --- | --- |
| Jan | 10% of soy hulls | 7.45 | 2594 |
| Feb | 10% of soy hulls | 7.36 | 2906 |
| Mar | 10% of soy hulls | 8.65 | 2321 |
| Apr | 10% of soy hulls | 11.57 | 455 |
| May | 10% of soy hulls | 14.59 | 336 |
| Jun | 10% of soy hulls | 17.05 | 619 |
| Jul | 10% of soy hulls | 19.01 | 913 |
| Aug | 10% of soy hulls | 19.00 | 1130 |
| Sep | 10% of soy hulls | 16.80 | 1305 |
| Oct | 10% of soy hulls | 13.65 | 1638 |
| Nov | 10% of soy hulls | 10.77 | 1971 |
| Dec | 10% of soy hulls | 8.35 | 2304 |
| Jan | 10% of wheat | 7.45 | 2074 |
| Feb | 10% of wheat | 7.36 | 2323 |
| Mar | 10% of wheat | 8.65 | 1856 |
| Apr | 10% of wheat | 11.57 | 363 |
| May | 10% of wheat | 14.59 | 269 |
| Jun | 10% of wheat | 17.05 | 495 |
| Jul | 10% of wheat | 19.01 | 730 |
| Aug | 10% of wheat | 19.00 | 903 |
| Sep | 10% of wheat | 16.80 | 1043 |
| Oct | 10% of wheat | 13.65 | 1309 |
| Nov | 10% of wheat | 10.77 | 1575 |
| Dec | 10% of wheat | 8.35 | 1842 |
