# Supplementary material for: Estimating the influence of dietary composition and management on nutrient intake and excretion and methane emission in different pig categories
Source: PLoS One. 2025 May 28;20(5):e0323024. doi: 10.1371/journal.pone.0323024 (PMC12119022; doi:10.1371/journal.pone.0323024)
Supplement: S1 Table — (ZIP) [file pone.0323024.s001.zip › Supporting information_Table_1.docx]

**S1 Table. Diet composition used for estimation of nutrient intake and excretion and CH_4_ emissions in weaned pigs (% of DM)**

|  | Average Danish diet | 5% of sugar beet | 5% of wheat bran | 10% of oats | 10% of wheat |
| --- | --- | --- | --- | --- | --- |
| Barley | 20.00 | 20.00 | 20.00 | 20.00 | 10.00 |
| Wheat | 56.11 | 51.17 | 51.17 | 46.16 | 66.10 |
| Oats | 0.00 | 0.00 | 0.00 | 10.00 | 0.00 |
| Sugar beet pulp | 0.00 | 5.00 | 0.00 | 0.00 | 0.00 |
| Wheat bran | 0.00 | 0.00 | 5.00 | 0.00 | 0.00 |
| Soybean meal, toasted | 14.00 | 14.00 | 14.00 | 14.00 | 14.00 |
| Potato protein | 2.50 | 2.50 | 2.50 | 2.50 | 2.50 |
| Fish meal | 1.00 | 1.00 | 1.00 | 1.00 | 1.00 |
| Vegetable oil | 1.80 | 1.80 | 1.80 | 1.80 | 1.80 |
| L-lysine (70%) | 1.10 | 1.10 | 1.10 | 1.10 | 1.10 |
| DL- methionine | 0.23 | 0.23 | 0.23 | 0.23 | 0.23 |
| L- threonine | 0.30 | 0.30 | 0.30 | 0.30 | 0.30 |
| L- tryptophan | 0.09 | 0.09 | 0.09 | 0.09 | 0.09 |
| L- valine | 0.15 | 0.15 | 0.15 | 0.15 | 0.15 |
| Monocalcium phosphate | 0.30 | 0.30 | 0.20 | 0.30 | 0.30 |
| Calcium carbonate (36% calcium) | 1.52 | 1.46 | 1.56 | 1.47 | 1.53 |
| Salt | 0.70 | 0.70 | 0.70 | 0.70 | 0.70 |
| Vitamin and mineral supplement | 0.20 | 0.20 | 0.20 | 0.20 | 0.20 |
| Nutrient composition |  |  |  |  |  |
| FEsv /100 kg feed | 112 | 108 | 109 | 109 | 113 |
| FEso/ 100 kg feed | 110 | 108 | 108 | 108 | 111 |
| Crude protein, g/kg | 178 | 177 | 180 | 177 | 179 |
| AA composition, g/kg |  |  |  |  |  |
| Lysine | 14.6 | 14.5 | 14.6 | 14.6 | 14.4 |
| Methionine | 5.0 | 4.9 | 5.0 | 5.0 | 5.0 |
| Cysteine | 3.0 | 2.9 | 3.0 | 3.0 | 3.0 |
| Threonine | 9.2 | 9.2 | 9.3 | 9.2 | 9.2 |
| Tryptophan | 3.1 | 3.0 | 3.1 | 3.1 | 3.1 |
| Isoleucine | 6.7 | 6.7 | 6.8 | 6.7 | 6.7 |
| Leucin | 12.4 | 12.3 | 12.6 | 12.5 | 12.5 |
| Histidine | 4.0 | 4.0 | 4.1 | 4.0 | 4.0 |
| Phenylalanine | 8.0 | 7.9 | 8.1 | 8.0 | 8.1 |
| Phenylalanine + Tyrosine | 5.8 | 5.8 | 5.8 | 5.8 | 5.8 |
| Valine | 9.6 | 9.6 | 9.7 | 9.6 | 9.5 |
| Calcium, g/kg | 7.0 | 7.0 | 7.0 | 6.8 | 7.0 |
| Total phosphorous, g/kg | 4.0 | 3.9 | 4.0 | 4.0 | 4.0 |
| Digestible phosphorous, g/kg | 2.5 | 2.4 | 2.5 | 2.5 | 2.5 |
