# Supplementary material for: Estimating the influence of dietary composition and management on nutrient intake and excretion and methane emission in different pig categories
Source: PLoS One. 2025 May 28;20(5):e0323024. doi: 10.1371/journal.pone.0323024 (PMC12119022; doi:10.1371/journal.pone.0323024)
Supplement: S1 Table — (ZIP) [file pone.0323024.s001.zip › Supporting information_Table_2.docx]

**S2 Table. Diet composition used for estimation of nutrient intake and excretion and CH_4_ emissions in grower-finisher pigs (% of DM)**

|  | Average Danish diet | 5% of sugar beet | 5% of wheat bran | 10% of oats | 10% of wheat |
| --- | --- | --- | --- | --- | --- |
| Barley | 27.00 | 27.00 | 27.00 | 27.00 | 17.00 |
| Wheat | 40.43 | 35.50 | 35.51 | 30.43 | 50.43 |
| Rye | 10.00 | 10.00 | 10.00 | 10.00 | 10.00 |
| Oats | 0.00 | 0.00 | 0.00 | 10.00 | 0.00 |
| Sugar beet pulp | 0.00 | 5.00 | 0.00 | 0.00 | 0.00 |
| Wheat bran | 0.00 | 0.00 | 5.00 | 0.00 | 0.00 |
| Soybean meal- toasted | 16.70 | 16.70 | 16.70 | 16.70 | 16.70 |
| Vegetable oil | 0.80 | 0.80 | 0.80 | 0.80 | 0.80 |
| L-lysine (70%) | 0.45 | 0.45 | 0.45 | 0.45 | 0.45 |
| DL- methionine | 0.07 | 0.07 | 0.07 | 0.07 | 0.07 |
| L- threonine | 0.20 | 0.20 | 0.20 | 0.20 | 0.20 |
| L- tryptophan | 0.30 | 0.30 | 0.20 | 0.30 | 0.30 |
| L- valine | 1.45 | 1.38 | 1.47 | 1.45 | 1.45 |
| Monocalcium phosphate | 0.40 | 0.40 | 0.40 | 0.40 | 0.40 |
| Calcium carbonate (36% calcium) | 0.20 | 0.20 | 0.20 | 0.20 | 0.20 |
| Nutrient composition |  |  |  |  |  |
| FEsv /100 kg feed | 107 | 103 | 104 | 104 | 108 |
| FEso/ 100 kg feed | 106 | 103 | 104 | 103 | 107 |
| Crude protein, g/kg | 160 | 159 | 163 | 160 | 162 |
| AA composition, g/kg |  |  |  |  |  |
| Lysine | 9.9 | 10.0 | 10.1 | 10.0 | 9.9 |
| Methionine | 3.1 | 3.0 | 3.1 | 3.1 | 3.1 |
| Cysteine | 2.9 | 2.8 | 2.9 | 2.9 | 2.9 |
| Threonine | 7.6 | 7.6 | 7.7 | 7.6 | 7.6 |
| Tryptophan | 2.1 | 2.0 | 2.1 | 2.1 | 2.1 |
| Isoleucine | 6.1 | 6.1 | 6.2 | 6.1 | 6.1 |
| Leucin | 11.2 | 11.1 | 11.3 | 11.2 | 11.3 |
| Histidine | 3.8 | 3.9 | 3.9 | 3.8 | 3.9 |
| Phenylalanine | 7.4 | 7.4 | 7.5 | 7.4 | 7.5 |
| Phenylalanine + Tyrosine | 5.0 | 5.1 | 5.1 | 5.1 | 5.0 |
| valine | 7.3 | 7.3 | 7.5 | 7.4 | 7.3 |
| Calcium, g/kg | 6.6 | 6.6 | 6.6 | 6.6 | 6.6 |
| Total phosphorous, g/kg | 4.1 | 4.0 | 4.1 | 4.0 | 4.1 |
| Digestible phosphorous, g/kg | 2.5 | 2.5 | 2.5 | 2.5 | 2.5 |
