# Supplementary material for: Estimating the influence of dietary composition and management on nutrient intake and excretion and methane emission in different pig categories
Source: PLoS One. 2025 May 28;20(5):e0323024. doi: 10.1371/journal.pone.0323024 (PMC12119022; doi:10.1371/journal.pone.0323024)
Supplement: S1 Table — (ZIP) [file pone.0323024.s001.zip › Supporting information_Table_3.docx]

**S3 Table. Diet composition used for estimation of nutrient intake and excretion and CH_4_ emissions in lactating sows (% of DM)**

|  | Average Danish diet | 5% of sugar beet | 5% of wheat bran | 10% of oats | 10% of wheat |
| --- | --- | --- | --- | --- | --- |
| Barley | 18.20 | 18.20 | 18.20 | 18.20 | 8.20 |
| Wheat | 49.92 | 45.04 | 45.02 | 40.30 | 59.80 |
| Rye | 10.00 | 10.00 | 10.00 | 10.00 | 10.00 |
| Oats | 0.00 | 0.00 | 0.00 | 10.00 | 0.00 |
| Sugar beet pulp | 2.50 | 7.50 | 2.50 | 2.50 | 2.50 |
| Wheat bran | 0.00 | 0.00 | 5.00 | 0.00 | 0.00 |
| Soy hulls | 0.00 | 0.00 | 0.00 | 0.00 | 0.00 |
| Soybean meal | 15.30 | 15.30 | 15.30 | 15.30 | 15.30 |
| Vegetable oil | 0.50 | 0.50 | 0.50 | 0.50 | 0.50 |
| L-lysine (70%) | 0.50 | 0.50 | 0.50 | 0.50 | 0.50 |
| DL- methionine | 0.10 | 0.10 | 0.10 | 0.10 | 0.10 |
| L- Threonine | 0.15 | 0.15 | 0.15 | 0.15 | 0.15 |
| Monocalcium phosphate | 0.80 | 0.80 | 0.65 | 0.80 | 0.85 |
| Calcium carbonate (36% calcium) | 1.45 | 1.35 | 1.50 | 1.45 | 1.50 |
| Salt | 0.38 | 0.36 | 0.38 | 0.00 | 0.40 |
| Vitamin and mineral supplement | 0.20 | 0.200 | 0.20 | 0.20 | 0.20 |
| Nutrient composition |  |  |  |  |  |
| FEsv /100 kg feed | 106 | 102 | 103 | 103 | 106 |
| FEso/100 kg feed | 105 | 103 | 103 | 103 | 105 |
| Crude protein, g/kg | 151 | 150 | 153 | 151 | 152 |
| AA composition, g/kg |  |  |  |  |  |
| Lysine | 9.6 | 9.7 | 9.8 | 9.7 | 9.6 |
| Methionine | 3.1 | 3.1 | 3.2 | 3.2 | 3.1 |
| Cysteine | 2.7 | 2.7 | 2.8 | 2.8 | 2.8 |
| Threonine | 6.6 | 6.7 | 6.7 | 6.7 | 6.6 |
| Tryptophan | 2.0 | 1.9 | 2.0 | 1.9 | 2.0 |
| Isoleucine | 5.6 | 5.6 | 5.7 | 5.7 | 5.6 |
| Leucin | 10.5 | 10.4 | 10.6 | 10.5 | 10.5 |
| Histidine | 3.6 | 3.6 | 3.7 | 3.6 | 3.6 |
| Phenylalanine | 6.9 | 6.8 | 7.0 | 6.9 | 6.9 |
| Phenylalanine + Tyrosine | 4.7 | 4.7 | 4.8 | 4.8 | 4.7 |
| Valine | 6.8 | 6.8 | 6.9 | 6.9 | 6.8 |
| Calcium, g/kg | 7.4 | 7.3 | 7.4 | 7.5 | 7.4 |
| Total phosphorous, g/kg | 4.9 | 4.8 | 4.9 | 5.0 | 4.9 |
| Digestible phosphorous, g/kg | 3.1 | 3.1 | 3.1 | 3.1 | 3.1 |
