# Supplementary material for: Estimating the influence of dietary composition and management on nutrient intake and excretion and methane emission in different pig categories
Source: PLoS One. 2025 May 28;20(5):e0323024. doi: 10.1371/journal.pone.0323024 (PMC12119022; doi:10.1371/journal.pone.0323024)
Supplement: S1 Table — (ZIP) [file pone.0323024.s001.zip › Supporting information_Table_4.docx]

**S4 Table. Diet composition used for estimation of nutrient intake and excretion and CH_4_ emissions in gestating sows (% of DM).**

|  | Average Danish diet | 10% of sugar beet | 10% of wheat bran | 10% of soy hulls | 10% of oats | 10% of wheat |
| --- | --- | --- | --- | --- | --- | --- |
| Barley | 47.20 | 47.20 | 47.20 | 47.20 | 47.20 | 37.20 |
| Wheat | 24.49 | 14.91 | 15.86 | 15.94 | 13.72 | 35.00 |
| Rye | 10.00 | 10.00 | 10.00 | 10.00 | 10.00 | 10.00 |
| Oats | 0.00 | 0.00 | 0.00 | 0.00 | 10.00 | 0.00 |
| Sugar beet pulp | 2.50 | 12.50 | 2.50 | 2.50 | 2.50 | 2.50 |
| Wheat bran | 0.00 | 0.00 | 10.00 | 0.00 | 0.00 | 0.00 |
| Soy hulls | 8.10 | 8.10 | 8.10 | 18.10 | 8.10 | 8.10 |
| Soybean meal, toasted | 5.20 | 5.00 | 4.00 | 4.00 | 6.00 | 4.60 |
| Vegetable oil | 0.50 | 0.50 | 0.50 | 0.50 | 0.50 | 0.50 |
| L-lysine (70%) | 0.00 | 0.00 | 0.00 | 0.00 | 0.00 | 0.05 |
| Monocalcium phosphate | 0.20 | 0.25 | 0.00 | 0.20 | 0.20 | 0.23 |
| Calcium carbonate (36% calcium) | 1.25 | 1.00 | 1.30 | 1.00 | 1.20 | 1.25 |
| Salt | 0.36 | 0.34 | 0.34 | 0.36 | 0.38 | 0.37 |
| Vitamin and mineral supplement | 0.20 | 0.20 | 0.20 | 0.20 | 0.20 | 0.20 |
| Nutrient composition |  |  |  |  |  |  |
| FEsv /100 kg feed | 98 | 91 | 93 | 88 | 94 | 99 |
| FEso/ 100 kg feed | 99 | 94 | 95 | 81 | 97 | 100 |
| Crude protein, g/kg | 107 | 108 | 107 | 102 | 109 | 106 |
| AA composition, g/kg |  |  |  |  |  |  |
| Lysine | 4.7 | 4.0 | 4.7 | 4.7 | 5.0 | 4.7 |
| Methionine | 1.7 | 1.5 | 1.7 | 1.6 | 1.7 | 1.7 |
| Cysteine | 2.2 | 3.2 | 2.3 | 2.1 | 2.3 | 2.2 |
| Threonine | 3.7 | 3.0 | 3.8 | 3.6 | 3.9 | 3.6 |
| Tryptophan | 1.4 | 1.1 | 1.4 | 1.3 | 1.4 | 1.4 |
| Isoleucine | 4.0 | 3.4 | 4.0 | 3.9 | 4.2 | 3.9 |
| Leucin | 7.4 | 6.3 | 7.3 | 7.0 | 7.7 | 7.3 |
| Histidine | 2.6 | 2.2 | 2.6 | 2.5 | 2.6 | 2.5 |
| Phenylalanine | 5.0 | 4.2 | 4.9 | 4.7 | 5.1 | 4.9 |
| Phenylalanine + Tyrosine | 3.4 | 7.2 | 3.4 | 3.4 | 3.6 | 3.4 |
| Valine | 5.1 | 4.2 | 5.2 | 5.0 | 5.3 | 5.0 |
| Calcium, g/kg | 5.9 | 5.5 | 5.8 | 5.4 | 5.8 | 5.9 |
| Total phosphorous, g/kg | 3.1 | 3.0 | 3.2 | 3.0 | 3.2 | 3.2 |
| Digestible phosphorous, g/kg | 1.9 | 1.9 | 1.9 | 1.8 | 1.9 | 1.9 |
